# Antitumour effect of polyoxomolybdates: induction of apoptotic cell death and autophagy in *in vitro* and *in vivo* models

**DOI:** 10.1038/sj.bjc.6604133

**Published:** 2007-12-18

**Authors:** A Ogata, H Yanagie, E Ishikawa, Y Morishita, S Mitsui, A Yamashita, K Hasumi, S Takamoto, T Yamase, M Eriguchi

**Affiliations:** 1 Chemical Resources Laboratory, Tokyo Institute of Technology, R1-21, 4259 Nagatsuta Midori-ku Yokohama, Tokyo 226-8503, Japan; 2 Cooperative Unit of Medicine and Engineering Research, The University of Tokyo Hospital, 7-3-1 Hongo Bunkyo-ku, Tokyo 113-8655, Japan; 3 Department of Nuclear Engineering and Management, School of Engineering, The University of Tokyo, 7-3-1 Hongo Bunkyo-ku, Tokyo 113-8655, Japan; 4 Department of Pathology, Graduate School of Medicine, The University of Tokyo, 7-3-1 Hongo Bunkyo-ku, Tokyo 113-8655, Japan; 5 Department of Molecular Health Sciences, Faculty of Pharmaceutical Sciences, Teikyo University, 1091-1 Suwarashi Sagamikochou Sagamihara, Kanagawa 229-0195, Japan; 6 Electro-Chemical and Cancer Research Institute, 5-45-6 Kokuryo-cho Chofu, Tokyo 182-0022, Japan; 7 Department of Cardiothoracic Surgery, Graduate School of Medicine, University of Tokyo, 7-3-1 Hongo Bunkyo-ku, Tokyo 113-8655, Japan; 8 Department of Microbiology, Showa University School of Pharmaceutical Sciences, 1-5-8 Hatanodai Shinagawa-ku, Tokyo 142-8555, Japan

**Keywords:** polyoxomolybdate, apoptosis, autophagy

## Abstract

Polyoxomolybdates (PMs) as discrete molybdenum-oxide cluster anions have been investigated in the course of study of their medical applications. Here, we show the significant antitumour potency of the polyoxomolybdate [Me_3_NH]_6_[H_2_Mo^V^_12_O_28_(OH)_12_(Mo^VI^O_3_)_4_]·2H_2_O (PM-17), which is a photo-reduced compound of [NH_3_Pr^i^]_6_[Mo_7_O_24_]·3H_2_O. The effect of PM-17 on the growth of cancer cell lines and xenografts was assessed by a cell viability test and analysis of tumour expansion rate. Morphological analysis was carried out by Hoechst staining, flow-cytometric analysis of Annexin V staining, terminal deoxynucleotidyl transferase-mediated ‘nick-end’ labelling staining, and electron-microscopic analysis. Activation of autophagy was detected by western blotting and fluorescence-microscopic analysis of the localisation of GFP-LC3 in transfected tumour cells. PM-17 inhibited the growth of human pancreatic cancer (AsPC-1) xenografts in a nude mice model, and induced morphological alterations in tumour cells. Correspondingly, PM-17 repressed the proliferation of AsPC-1 cells and human gastric cancer cells (MKN45) depending on the dose *in vitro*. We observed apoptotic patterns as the formation of apoptotic small bodies and translocation of phosphatidylserine by Hoechst staining and flow-cytometric analysis following Annexin V staining, and in parallel, autophagic conformation by the formulation of autophagosomes and localisation of GFP-LC3 by electron- and fluorescence-microscopic analysis.

Pancreatic cancer is an aggressive form of cancer, and has one of the poorest outcomes of all cancers (Jemal *et al*, 2005; Tani *et al*, 2006). Less than 10% of newly diagnosed pancreatic cancer patients achieve 5-year survival (Fakih, 2006). Although the clinical investigation of several antitumour agents has been conducted with a principal focus on fluorouracil, no agent has been found to suppress tumour growth. Also, there is no obvious improvement in the results of treatment with multiple drug therapy (Kelsen *et al*, 1991; Topham *et al*, 1991; Rougier *et al*, 1993; Nose *et al*, 1999) or the biochemical modulation of fluorouracil (Pazdur *et al*, 1992; Louvet *et al*, 1993; Weinerman and MacCormick, 1994; Bernhard *et al*, 1995). Gemcitabine, a nucleoside analogue, is efficacious in relieving the symptoms such as algia or general state in the case of nonscaling down of tumours (Casper *et al*, 1994). Furthermore, since the gemcitabine group has a beneficial effect on survival rate compared with the fluorouracil group, gemcitabine is the drug of choice for progressive pancreatic cancer at present. However, in recent findings, there is a significant toxic effect of gemcitabine combined with radiation in the treatment of advanced pancreatic cancer (McGinn *et al*, 2001, 2002; Wolff *et al*, 2001; Crane *et al*, 2002). In addition, since existing antipancreatic cancer drugs have no eradicative potency, it is necessary to find medicines that have new mechanisms of antitumour activity.

Dramatic improvement has been made in the treatment of patients by modern chemotherapeutic protocols, which are based on the dose-dependent antitumour activity of cisplatin (CDDP). However, the excellent antitumour activity of CDDP is accompanied by strong toxic effects. From chemical analogy with CDDP, antitumour active organometallic compounds such as titanocene dichloride, copper (I), silver (I), and gold (I or III) tetrahedral diphosphine complexes, and organosilicone compounds such as trimethylsilylethylthioethylamine and its derivatives, have been found (Fujita *et al*, 1990). In the course of examination of the biological effects of polyoxometalates, inorganic polymers of metal oxide, significant antitumour effects of polyoxomolybdates were found against conventional murine tumours (Fujita *et al*, 1992). [NH_3_Pr^i^]_6_[Mo_7_O_24_]·3H_2_O (PM-8) was found to exhibit potent antitumour activity against MX-1 human breast cancer, Meth-A sarcoma, and MM46 adenocarcinoma *in vivo* (Yamase *et al*, 1988). Recent work from our laboratory showed that the efficacy of PM-8 has been recognised also for both AsPC-1 human pancreatic cancer and MKN45 human gastric cancer, which results from activation of the apoptotic pathway (Ogata *et al*, 2005; Mitsui *et al*, 2006; Yanagie *et al*, 2006). It has been proposed that PM-8 could be preferentially taken into tumour cells and converted into an approximately 10-fold more toxic species, produced through biological reduction probably in the mitochondrial system. This would inhibit ATP generation, and such a significant difference in toxicity between PM-8 and its reduced species would lead to a tumour-selective inhibition. This is based on the enhanced metabolism in tumour cells, since the high molecular weight of the reduced species (as a condensed species) would result in a longer stay in the tumour cells (Yamase, 2005).

In this study, we show noticeable antitumour activity of PM-17 (=[Me_3_NH]_6_[H_2_Mo^V^_12_O_28_(OH)_12_(Mo^VI^O_3_)_4_]·2H_2_O), one of the reduced species of PM-8, in the treatment of AsPC-1 cells and (or) MKN45 cells *in vitro* and *in vivo*. Furthermore, we aimed to clarify the mechanism of the anticancer effects of these compounds.

## MATERIALS AND METHODS

### PM-8 and PM-17

PM-8 was synthesised according to the published procedure (Yamase and Ikawa, 1977). PM-17 was prepared as follows: an aqueous solution (20 ml) of PM-8 (0.3 g, 0.2 mmol) in a Pyrex tube was irradiated for 4 days using a 500-W superhigh-pressure mercury/xenon lamp. ^i^PrNH_2_ was added dropwise to the dark brown-coloured photolyte to adjust the pH to 8.8, then added [Me_3_NH]Cl (1 g, 10.4 mmol), and followed by cooling at 4°C. Reddish-brown rod crystals of the photo-reduced product were precipitated after 1–2 weeks and isolated by filtration. Yield: 64.3 mg, 25.9% based on Mo. Calcd: N 3.03, C 7.78, H 2.83, Mo 55.3%; found: N 3.42, C 8.16, H 2.56, Mo 53.8%. Manganometric redox titration showed the presence of 11.6±0.1 Mo^V^ centres in PM-17. Together with the X-ray crystallographic results (Yamase and Ishikawa), the elemental analysis enabled us to formulate PM-17 as given.

### Animals and cell lines

Female nude mice (BALB/c, nu/nu) were purchased from Japan SLC Inc. The experiments were performed with the permission of the Animal Ethics Committee of The University of Tokyo in accordance with the Declaration of Helsinki. At the start of the experiments, the mice were 7 weeks old and were kept under controlled, specific pathogen-free conditions (temperature 23±2°C, relative humidity 55±5% and 12 h/12 h light/dark cycle) in isolation. An AsPC-1 human pancreatic cancer cell line and a MKN45 human gastric cancer cell line were used in this study, and were maintained in RPMI 1640 medium (GIBCO, Carlsbad, CA, USA) supplemented with 10% FBS (CLONTECH Laboratories, Mountain View, CA, USA), 100 U ml^−1^ of penicillin G, and 100 U ml^−1^ streptomycin in a 5% CO_2_ humidified atmosphere at 37°C.

### Tumour growth inhibition test

To estimate the antitumour effects of PM-17 on the proliferation of AsPC-1 cells *in vivo*, 2 × 10^6^ cells were transplanted into the back of nude mice. After 10 days, intratumoral injections of PM-17 (125*μ*g or 500 *μ*g dissolved in 100 *μ*l saline) were performed for 10 days with a 2-day intermission on day 6. The control mice were treated with 100 *μ*l saline per day under the same conditions used for the animals treated with PM-17. The tumour volume was determined using the two principal perpendicular diameters as *V*=1/2 × length (mm) × (width (mm))^2^.

### Antibodies and chemicals

Antibodies were obtained from the following commercial sources: anti-MAP LC3 (Santa Cruz Biotechnology Inc., Santa Cruz, CA, USA); anti-*β*-actin antibody (SIGMA-Aldrich, Saint Louis, MO, USA). Horseradish peroxidase-conjugated Streptavidin was purchased from Amersham Biosciences (Buckinghamshire, UK). PM-17 was prepared by the chemical synthesis group in our laboratory.

### Cell viability assay

To determine the effect of PM-17 on tumour cells, we assessed cell viability after treatment using the trypan blue dye exclusion assay (Takeuchi *et al*, 2005). AsPC-1 and MKN45 in exponential growth phase were harvested, seeded at 1.5 × 10^5^ cells per 6 cm cell culture dish (5 ml), and incubated at 37°C in 5% CO_2_ in air overnight. The cells were then incubated for 24 h with or without PM-17 in graduated concentrations from 0 to 230 *μ*g ml^−1^ (AsPC-1) and from 0 to 60 *μ*g ml^−1^ (MKN45). After the cells were collected with a scraper and stained with trypan blue, the viable cells were counted using a haemocytometer. Considering the viability of untreated cells (the control) as 100%, we calculated survival fractions from the mean cell viability of the treated cells.

### Quantification of apoptosis *in situ*

Analysis of DNA fragmentation of the lamellae of the tumour by terminal deoxynucleotidyl transferase-mediated ‘nick-end’ labelling (TUNEL) was done using a POD labelling *in situ* cell death detection kit (Roche, Basel, Switzerland). DNA fragmentation was detected by enzyme reaction of labelled POD with DAB substrate (Roche).

### Detection of apoptotic small bodies

One million cells growing in 25 cm^2^ culture flasks were exposed to PM-17 at concentrations of 175 *μ*g ml^−1^ (AsPC-1) and 40 *μ*g ml^−1^ (MKN45) for 24 h. The cells were harvested and centrifuged at 200 **g** for 10 min before being washed and resuspended in PBS containing 1 mM Hoechst dye 33342 (SIGMA-Aldrich). The apoptotic cells were visualised by fluorescence microscopy (KEYENCE CORPORATION, Osaka, Japan).

### Detection of DNA ladder

Cells growing in 10-cm diameter culture dishes were treated with PM-17 at concentrations of 0 or 175 *μ*g ml^−1^ (AsPC-1) and 0 or 40 *μ*g ml^−1^ (MKN45) for 24 h. Following centrifugation at 200 **g**, the harvested cells were lysed with DNA extraction buffer (10 mM Tris-HCl (pH 7.4), 10 mM EDTA, 0.5% Triton X-100) and incubated at 4°C for 10 min. After centrifugation at 16 000 **g** for 5 min, the cell lysate was treated with 500 *μ*g ml^−1^ DNase-free RNase at 37°C for 1 h, followed by treatment with 500 *μ*g ml^−1^ proteinase K at 50°C for 30 min. Electrophoresis in 1% agarose gels was then performed and DNA ladders were visualised by UV illumination after staining with ethidium bromide.

### Quantification of disruption of cytoplasmic membrane

The translocation of phosphatidylserine from the inner part of the plasma membrane to the outer layer was detected with Annexin-V-FLUOS staining; the disruption of the cytoplasmic membrane was analysed by the formulation of PI–DNA complexes by flow cytometry with a FAC Sort FCM machine (Beckman Coulter, Fullerton, CA, USA). The experiments were performed with Annexin-V-FLUOS followed by staining with the Roche kit.

### Measurement of caspase-3 activity

Activity of caspase-3 was measured by Caspase-Glo™ 3/7 Assay kit (Promega Corporation, Madison, WI, USA). The assay provides a proluminescent caspase-3/7 substrate, which contains the tetrapeptide sequence DEVD. Luminescence is proportional to the amount of caspase activity present. The experimental procedures followed the manufacturer's instructions. We measured the intensity of luminescence with a Mithras LB 940 microplate reader (Berthold Technologies, Bad Wildbad, Germany).

### Western blot analysis

Two million cells were cultured in sterile 10 cm dishes in 10% FCS/RPMI 1640 culture medium overnight. Next day, the cells were exposed to experimental medium with or without PM-17 (MKN45, 40 *μ*g ml^−1^; AsPC-1, 175 *μ*g ml^−1^) for 24, 48, or 72 h. The cultured cells were centrifuged at 200 **g** for 5 min at 4°C and washed in ice-cold PBS.

After centrifugation, the supernatant was removed and the cell pellet was resuspended in 0.1 ml lysis buffer (PBS, 1% Triton X-100, 1% Nonidet P-40), and incubated at 4°C for 30 min. The cells suspended in the buffer were centrifuged at 10 000 **g** for 10 min at 4°C, and then the supernatant (containing the total protein fraction) was carefully transferred to a new tube. Each sample, containing equal amounts of protein (80 *μ*g), was mixed 1 : 3 (v v^−1^) with sample buffer (Wako Pure Chemical Industries Ltd, Osaka, Japan) containing 20% 2-mercaptoethanol and boiled for 5 min. Samples containing identical quantities of protein were subjected to SDS–PAGE (4–20% gradient gel) together with biotinylated SDS–PAGE standards (Bio-Rad, Hercules, CA, USA). Electrophoresis was performed for almost 1 h at 40 mA. After electrophoresis, the separated proteins were electroblotted onto PVDF membranes (Millipore Corporation, Bellerica, MA, USA) for 60 min at 20 V. The membranes were blocked for 1 h with blocking solution (Dainippon Sumitomo Pharma, Osaka, Japan) dissolved in PBS-T (0.05% Tween 20 in PBS), then incubated with the primary antibodies diluted with blocking solution at 4°C. The dilution rates of the antibodies were as follows: anti-MAP LC3 antibody 1 : 200 and anti-*β*-actin antibody 1 : 3000. The following day, the membranes were rinsed three times for 10 min each in PBS-T at room temperature and incubated with HRP-labelled secondary antibodies (1 : 3000) and HRP-conjugated Streptavidin (1 : 3000) (GE Healthcare UK Ltd, Buckinghamshire, UK) diluted in blocking solution for 1 h at room temperature. The membranes were then washed three times for 10 min each in PBS-T and reacted with ECL Plus Western Blotting Detection System (GE Healthcare UK Ltd). Chemiluminescence was analysed by ChemiDoc XRS (Bio-Rad).

### Transmission electron microscopy

Cells were fixed in a mixture of 2.5% glutaraldehyde in 0.1 M phosphate buffer, pH 7.4 for 2 h at 4°C, washed in 0.2 M sucrose/0.1 M phosphate buffer, pH 7.4, for 2 h, and postfixed with 1.5% osmium tetroxide in 0.1 M phosphate buffer for 1 h at room temperature. The material was dehydrated in an ethanol gradient (50, 70, 80, 90, and 100%) and propylene oxide, and embedded in Epon 812. The blocks were cut with a microtome and stained with 4% uranyl acetate and Reynolds solution. Samples were photographed in a JEOL JEM-1200 EX electron microscope at 80 kV.

### Transfection and fluorescence microscopy

MKN45 cells and AsPC-1 cells were transfected using lipofectamine 2000 reagent (Invitrogen, Carlsbad, CA, USA) and pGFP-LC3. One day before transfection, 2 × 10^5^ cells were spread in 24 well plates in 500 *μ*l growth medium. Then the transfection solution was prepared as follows: in one tube, 0.8 *μ*g of DNA was diluted in 50 *μ*l of serum-free medium and mixed gently, in the second tube, 2 *μ*l of lipofectamine 2000 reagent was diluted in 48 *μ*l of serum-free medium and incubated at room temperature for 5 min. After 5 min incubation, both solutions were carefully combined, mixed gently, and incubated at room temperature for 20 min. The cells were washed, the transfection mixture (100 *μ*l each) was added, and incubated at 37°C, under 5% CO_2_ humidified atmosphere for 6 h. Afterwards, the transfection mixture was replaced with growth medium. After 24 h, transfected cells were treated with or without PM-8 (MKN45, 900 *μ*g ml^−1^; AsPC-1, 1.65 mg ml^−1^) or PM-17 (MKN45; 40 *μ*g ml^−1^, AsPC-1; 175 *μ*g ml^−1^) for 24 h. The cells were resuspended in PBS and transferred onto glass slides. The fluorescence of GFP-labelled LC3 protein was observed by fluorescence microscopy (Keyence Corporation).

### Haematological examination and blood chemistry assay

BALB/c nude mice were transplanted with 2 × 10^6^ AsPC-1 cells into the back, and after 10 days, we carried out intratumoral injections of PM-17 (500 *μ*g or 1 mg per 100 *μ*l saline per day) for 10 days with a 2-day intermission on day 6. Control mice were treated with 100 *μ*l of saline per day under the same conditions used for animals treated with PM-17. Negative control mice received neither tumour transplantation nor intratumoral injection. After 24 h from the last intratumoral injection, mice were anaesthetised and blood samples taken. Whole blood samples were used for haematological tests. Blood sera were assessed for BUN, creatinine, total bilirubin, total protein, AST, ALT, and ALP.

### Statistical analysis

For the tumour cell inhibition assay *in vivo*, measurement of caspase-3 activity assay, haematological examination, and blood chemistry assay, statistical analysis was performed using *t*-test between control and samples treated with PM-17. *P*-values less than 0.05 indicate statistical significance.

## RESULTS

### PM-17 interferes with the growth of a solid tumour of AsPC-1 *in vivo*

**Figure 1:**
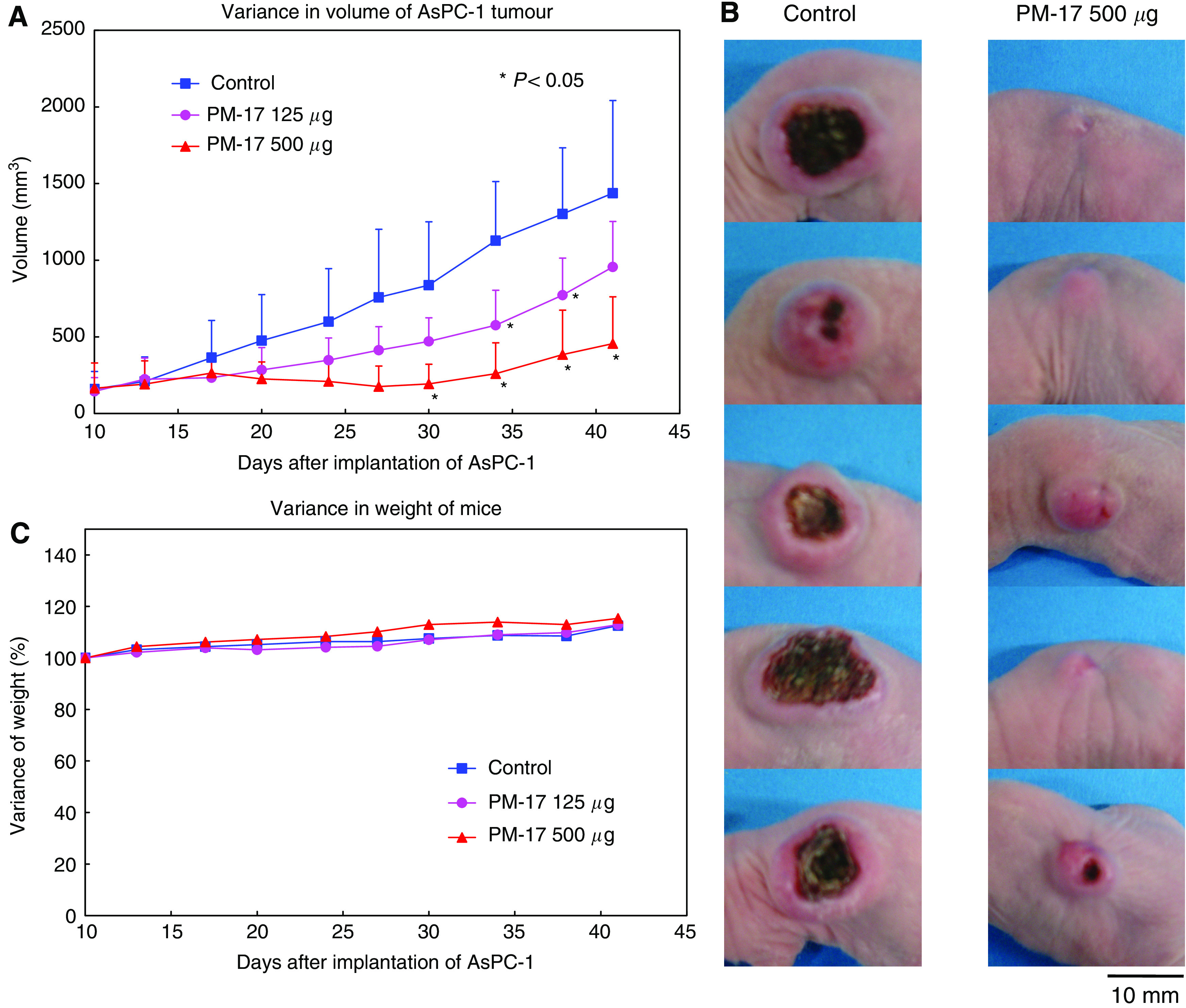
PM-17 inhibits tumour growth depending on the dose *in vivo*. (**A**) Variance of tumour volume. After 10 days implantation of 2 × 10^6^ AsPC-1 cells in BALB/c nude mice, these mice were treated with 0.9% NaCl solution (▪, *n*=5), PM-17 (125 *μ*g per 100 *μ*l) (•, *n*=5), and PM-17 (500 *μ*g per 100 *μ*l) (▴, *n*=5) intratumorally. The intratumoral injections were performed for 10 days with 2 days’ intermission on day 6. The tumour sizes were measured using a micrometre caliper. Points, mean of tumour volume of five mice per group; bars, s.d. ^*^P<0.05 compared with control. (**B**) Tumours of 41 days after implantation (left, saline control; right, PM-17 500 *μ*g per body treated). (**C**) Alteration of weight of tumour-bearing mice. Points, mean of percentage of body weight per group.

Timed measurements showed that PM-17 inhibited tumour growth at rates of 33.5 and 68.3% at doses of 125 *μ*g and 500 *μ*g per body per day (at 41 days after implantation of the tumour) compared with the controls (Figure 1A and B). In addition, there was no loss of body weight for mice injected with PM-17 in this experiment (Figure 1C). These results show that PM-17 inhibits the proliferation of AsPC-1 human pancreatic tumours in a dose-dependent manner *in vivo*.

### Inhibition of tumour cell growth with PM-17 *in vitro*

**Figure 2:**
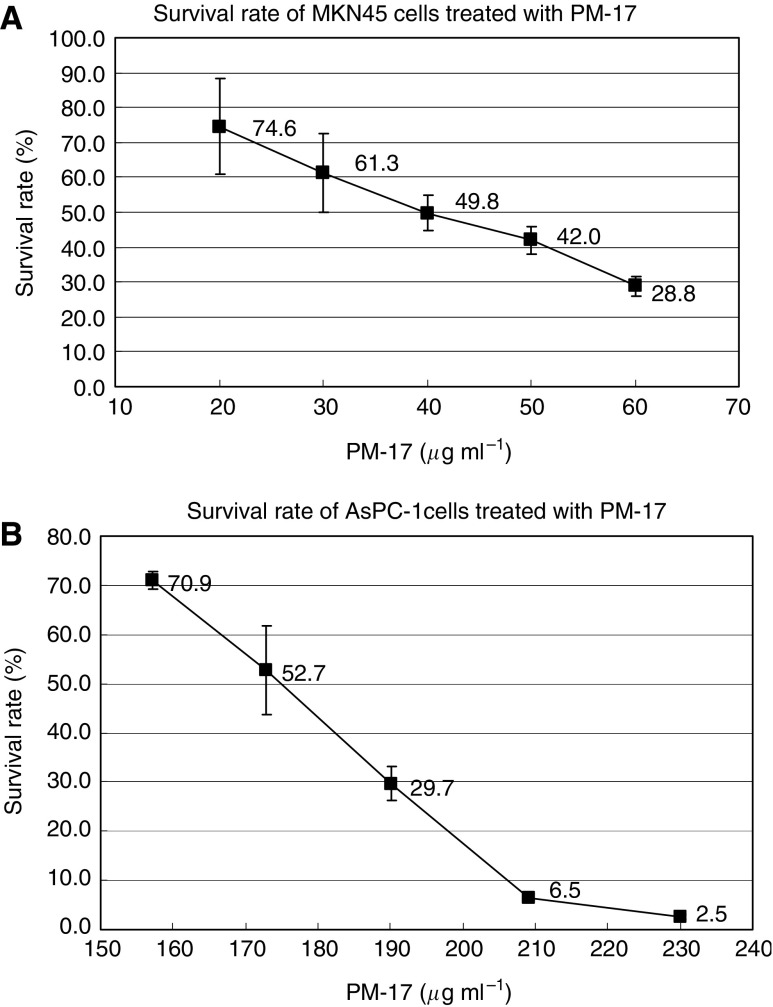
The effect of PM-17 treatment on MKN45 cell and AsPC-1 cell survival *in vitro*. MKN45 cells were treated with PM-17 at the concentrations of 0–60 *μ*g ml^−1^ for 24 h (**A**) and AsPC-1 cells were treated at the concentrations of 0–230 *μ*g ml^−1^ for 24 h (**B**). Points, percentage of living cell in three independent experiments; bars, s.d.

We examined the cytotoxic effects of PM-17 on the growth of AsPC-1 cells and MKN45 cells. These cells were suspended in RPMI 1640 culture medium containing a range of concentrations of PM-17. As shown in Figure 2, IC_50_ values of PM-17 against AsPC-1 cells and MKN45 cells were 175 and 40 *μ*g ml^−1^, respectively.

### Apoptotic changes of tumour cells treated with PM-17

**Figure 3:**
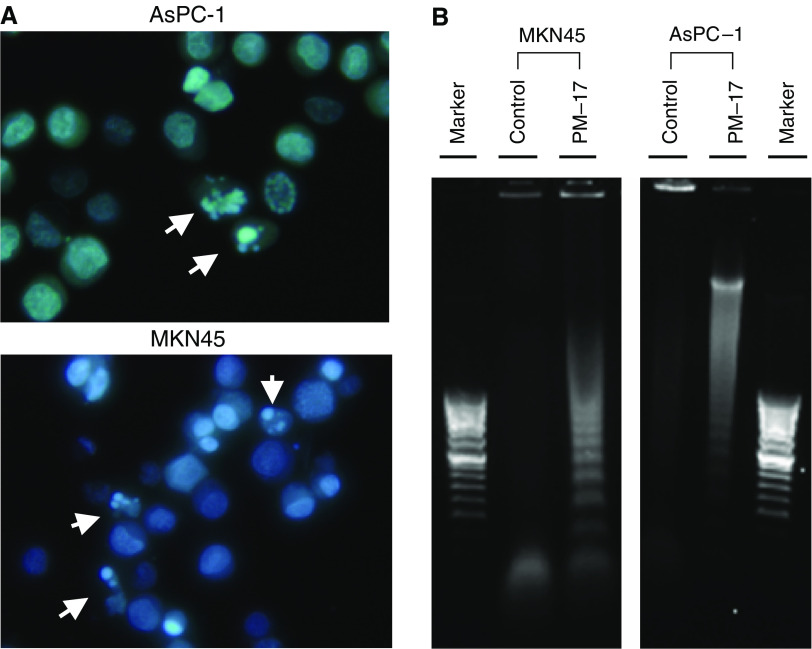

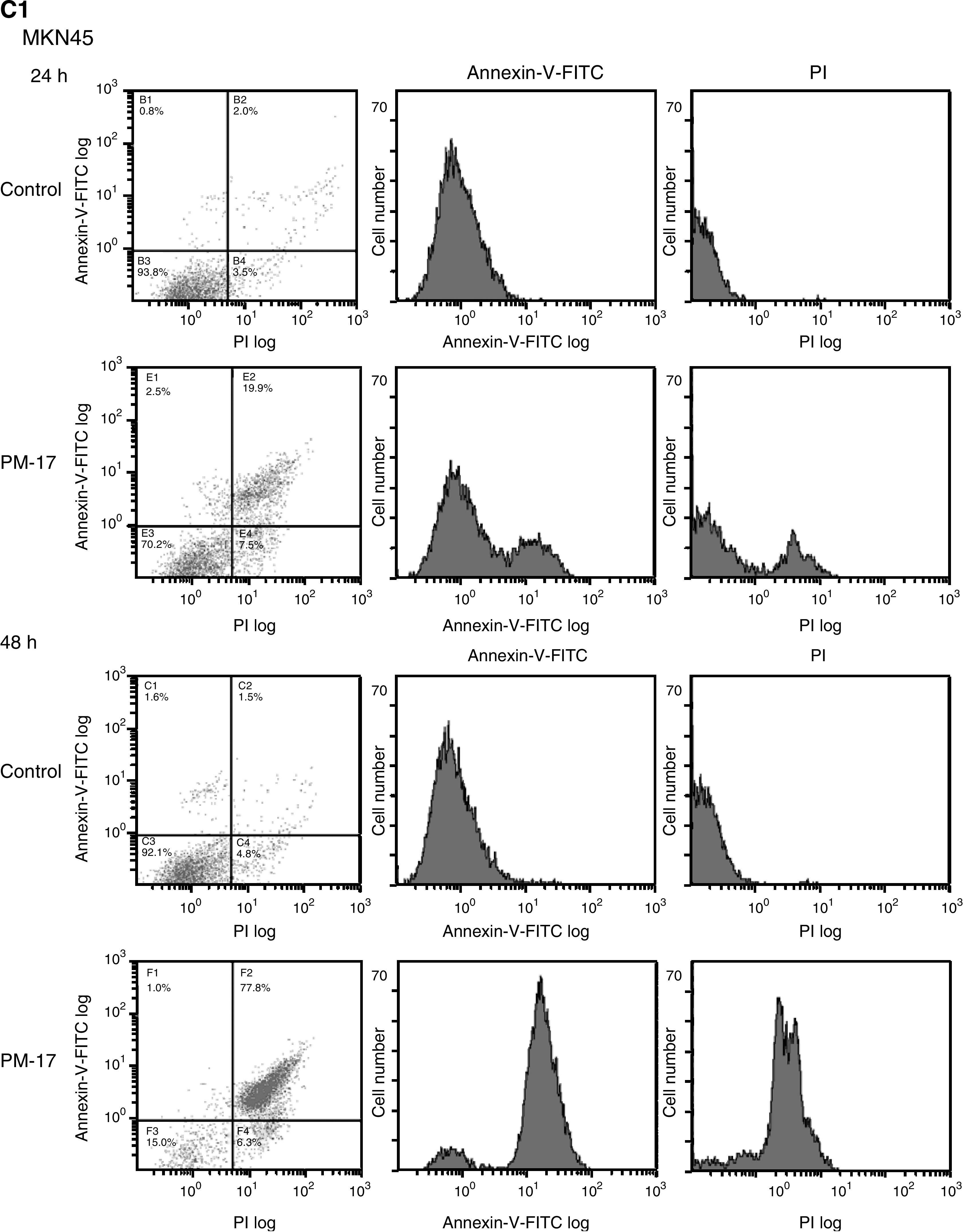

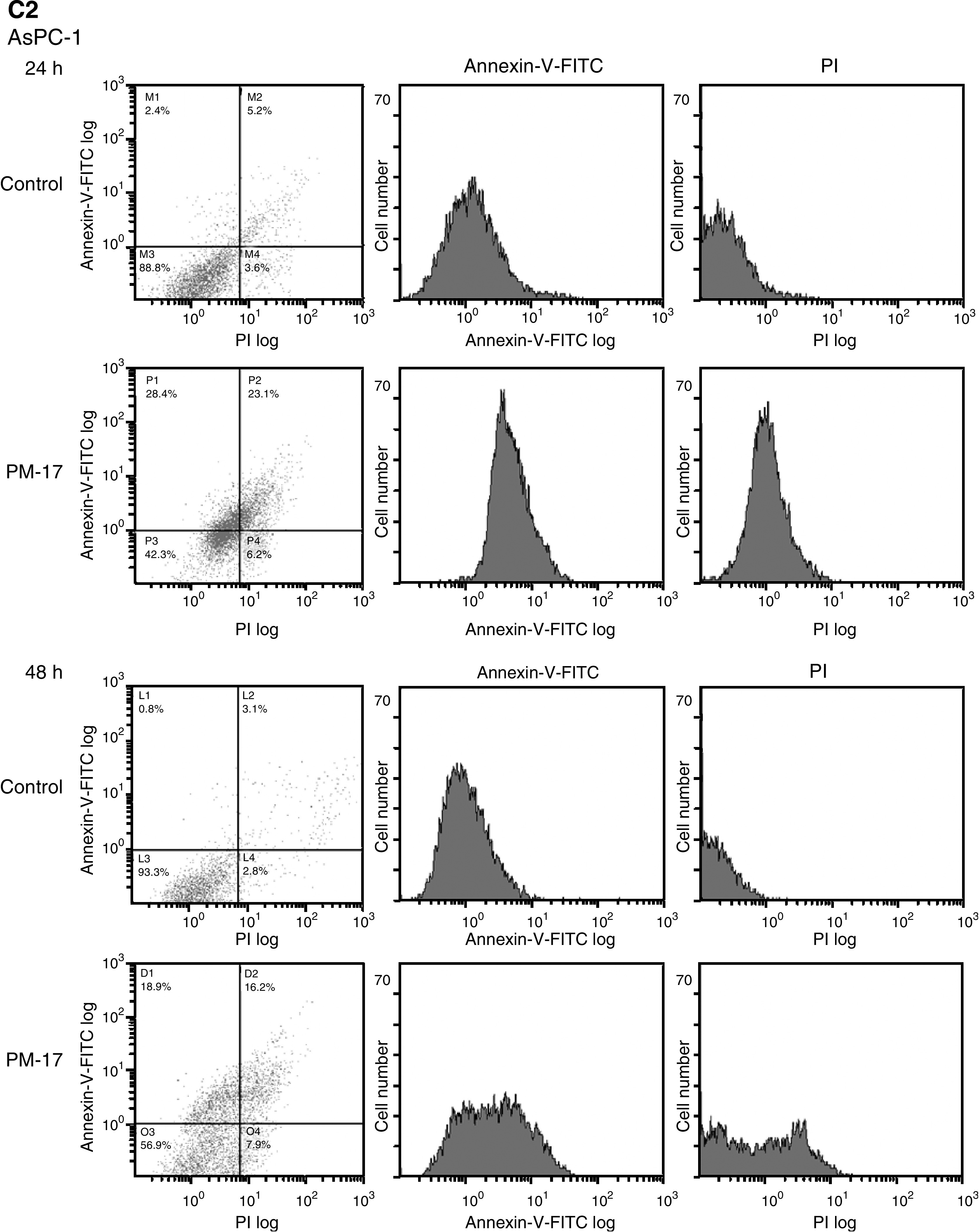

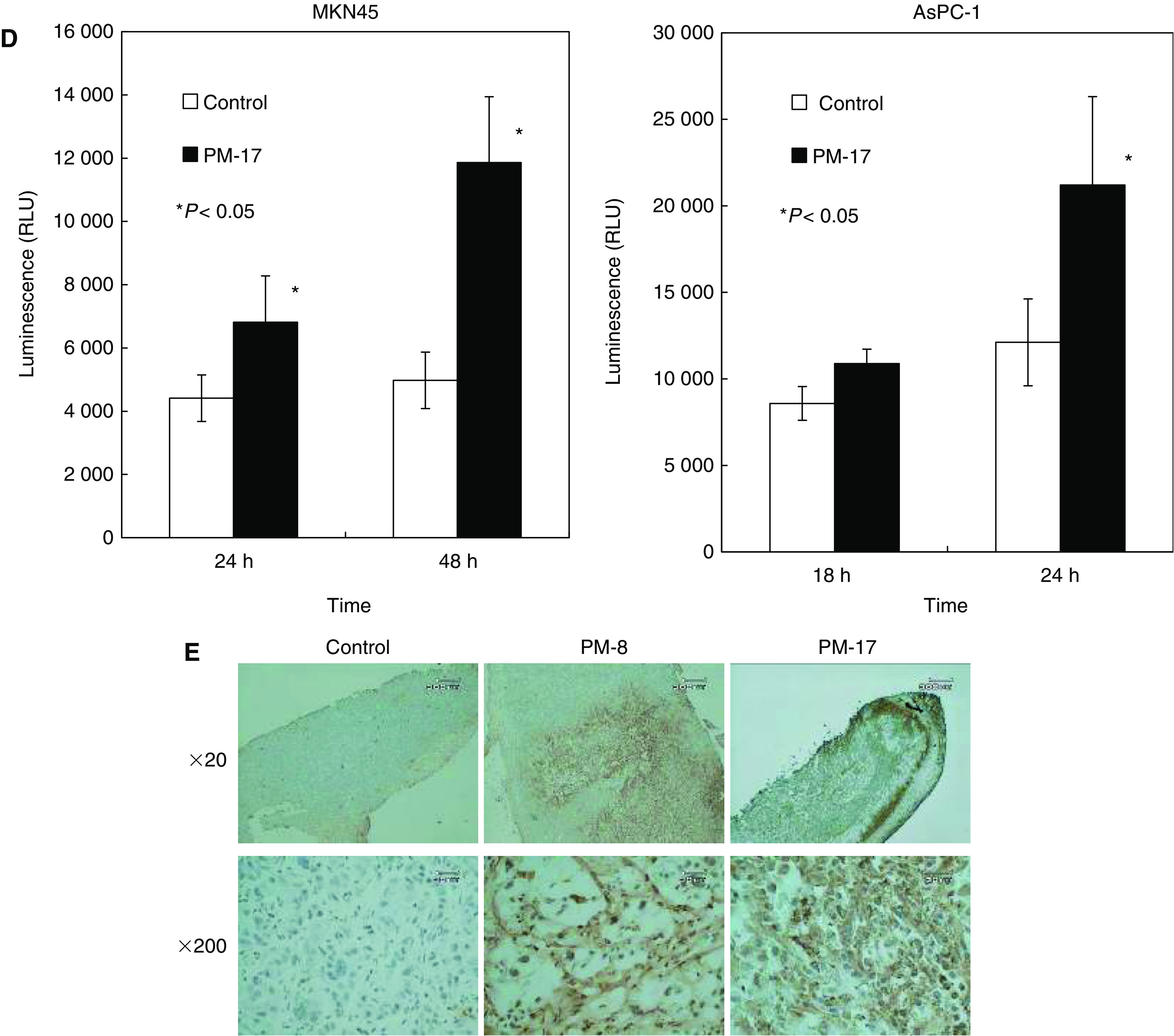
Apoptosis detection in tumour cells treated with PM-17. (**A**) Hoechst 33342 staining. The tumour cells treated with PM-17 at the IC_50_ concentrations (AsPC-1, 175 *μ*g ml^−1^; MKN45, 40 *μ*g ml^−1^) for 24 h were harvested and stained with Hoechst 33342. Arrows show apoptotic small bodies. (**B**) DNA ladder formation of AsPC-1 and MKN45 after 24 h treatment without or with IC_50_ of PM-17. DNA fragments derived from nontreated cells and treated cells were electrophoresed in agarose gel with 100 bp DNA ladder marker. (**C**) Flow-cytometric analysis by double-staining of Annexin-V-FLUOS and PI on AsPC-1 and MKN45 after 24 and 48 h treatment without or with IC_50_ of PM-17. (**D**) Activation of apoptosis signalling via caspase-3 activation in MKN45 cells and AsPC-1 cells treated with PM-17. The variation of caspase-3 activity with time was analysed by measurement of chemiluminescence of a proluminescent caspase-3/7 substrate. Columns, means of luminescence intensity response to activity of caspase-3 in three independent experiments; bars, s.d. ^*^P<0.05 compared with control, *n*=3. (**E**) TUNEL staining of tumours injected with PM-8 (4 mg per 100 *μ*l) or PM-17 (500 *μ*g per 100 *μ*l) or 100 *μ*l of 0.9% NaCl solution as control once for all. The observations were made with a wide or narrow field of view as shown in panels.

To investigate the mechanism of cell inhibition by PM-17, morphological analysis by Hoechst staining was performed to detect the formation of apoptotic small bodies, as shown in Figure 3A. Sharp DNA laddering was observed after treatment with PM-17 by agarose gel electrophoresis of cell DNA extracts, which allowed us to confirm the induction of apoptosis by PM-17 (Figure 3B).

Flow-cytometric analysis using Annexin V and PI staining was performed to determine the translocation of phosphatidylserine, disruption of cytoplasmic membranes, and produce quantitative results. Figure 3C shows that the ratio of apoptotic cells increased with the duration of PM-17 treatment: approximately 19.9% after 24 h and 77.8% after 48 h in MKN45 cells, and 16.2% after 24 h and 23.1% after 48 h in AsPC-1 cells.

The activation of caspase-3 was obtained in MKN45 cells and AsPC-1 cells treated with PM-17 as determined by Caspase-Glo™ 3/7 Assay. The substrate test used made it appear that caspase-3 was activated in both of the treated tumour cell lines (Figure 3D).

Moreover, TUNEL staining of thin sections of tumours after single injections of PM-8 or PM-17 showed the induction of cell degradation and DNA fragmentation (Figure 3E), and H&E staining showed that tumour cells treated with PM compounds led to hyalinisation by pathological observations. In the experiment, nude mice were injected with 2 × 10^6^ of AsPC-1 cells. After 10 days, we gave single injections of PM-8 (4 mg per 100 *μ*l saline) or PM-17 (500 *μ*g per 100 *μ*l saline) intratumorally. We then killed the mice after 24 h, eviscerated and fixed the tumours by freezing with O.C.T. Compound (Sakura Finetechnical Co. Ltd, Tokyo, Japan). Thin sections of tumours were stained with TUNEL stain. These results showed that PM-8 and PM-17 induced apoptosis of tumour cells *in vivo*.

### Morphological analysis and detection of autophagic features

**Figure 4:**
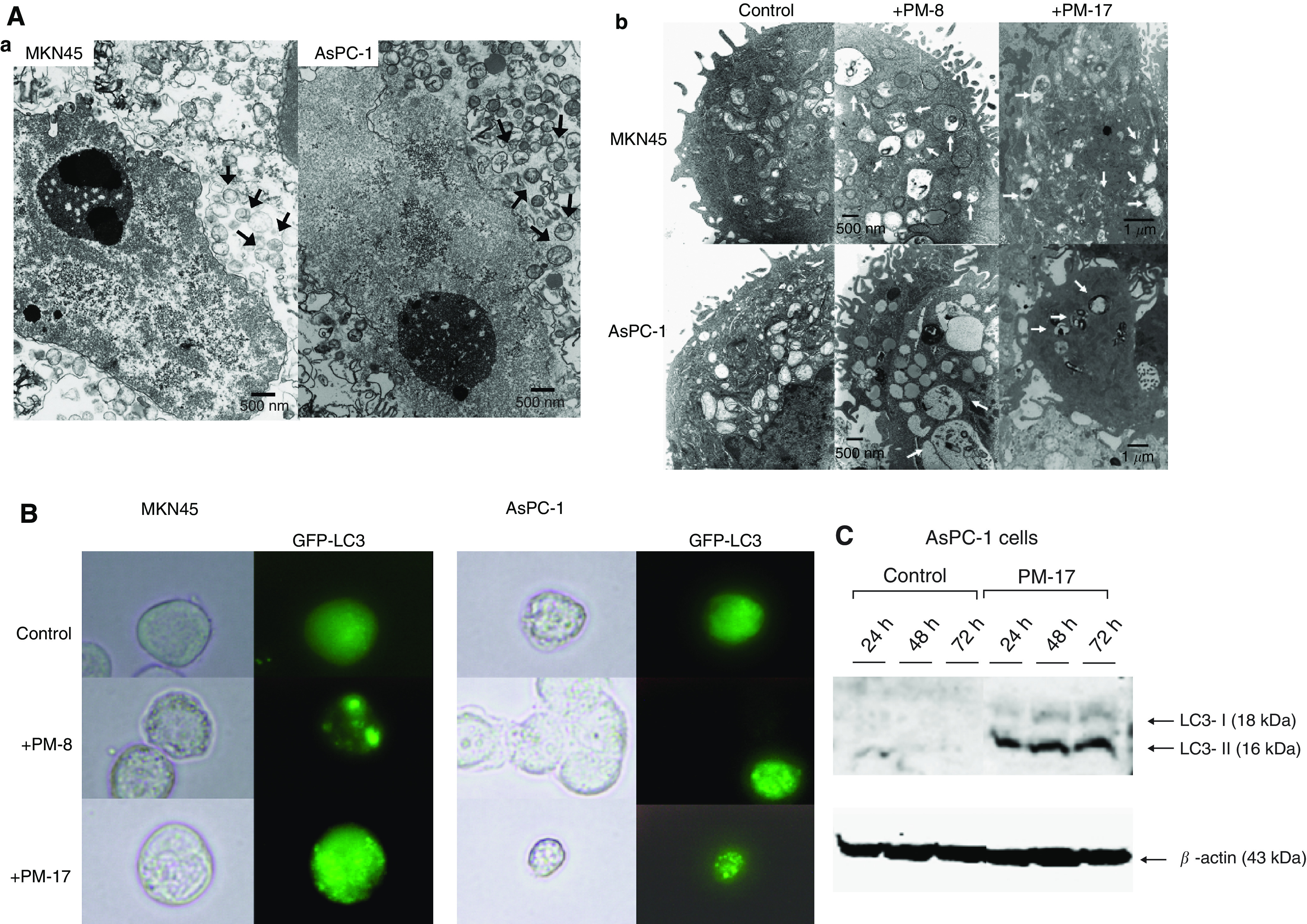
Morphological analysis and detection of autophagic feature. MKN45 cells were treated with PM-8 (900 *μ*g ml^−1^) or PM-17 (40 *μ*g ml^−1^), and AsPC-1 cells were treated with PM-8 (1.65 mg ml^−1^) or PM-17 (175 *μ*g ml^−1^). (**A**) Electron-microscopic analysis shows that mitochondria were disrupted. Inner constitution ((**a**), arrows) and appearance of quantity of autophagic vacuoles ((**b**), arrows) in MKN45 and AsPC-1 treated with IC_50_ of PM-8 or PM-17 for 24 h. (**B**) Subcellular localisation of GFP fusion LC3 protein in transfected MKN45 cells and AsPC-1 cells after treatment with or without IC_50_ of PM-8 or PM-17 for 24 h by fluorescent microscopy. (**C**) AsPC-1 cells treated with or without PM-17 (175 *μ*g ml^−1^) for 24, 48, and 72 h. The cells were spun and lysed, and each 80 *μ*g protein sample was loaded on SDS–PAGE gel and analysed by western blotting. Levels of LC3-I and -II expression were increased in cells treated with PM-17. Experiments were performed twice.

Electron-microscopic analysis shows that mitochondria had disrupted inner membranes in apoptotic MKN45 and AsPC-1 cells treated with PM-17 (Figure 4Aa, arrows). A number of vesicles, figurative autophagosomes, were formed (Figure 4Ab, arrows) in MKN45 and AsPC-1 cells treated with PM-8 and PM-17. Autophagic vacuoles contained cytosolic components and organelles.

With a view to determine if an autophagic pathway or another cell death pathway had been activated in the tumour cells or not, we observed the localisation of LC3 after transient transfection (of MKN45 cells and AsPC-1 cells) of a plasmid encoding GFP-LC3 24 h after treatment with PM-8 or PM-17 (Figure 4B). LC3-II is a truncated form of LC-3, and is necessary to combine with the seclusion membrane for wrapping around cytoplasm components fundamentally at random in the primary stage of autophagy (Takeuchi *et al*, 2005). The GFP-LC3 was expressed and localised on subcellular components, which were considered to be autophagic vacuoles, upon treatment with PM-8 or PM-17 in fluorescence microscopic observations. We also examined the expression of LC3-II. Western blot analysis showed that the expression of LC3-II protein increased in AsPC-1 cells treated with PM-17 (Figure 4C). These results suggest that treatment with PM-17 induces activation of two types of cell death pathway, not only apoptosis but also autophagy.

## DISCUSSION

Some polyoxomolybates such as PM-8, PM-17, PM-26, and PM-32 were found to exhibit antitumour activities against Co-4 human colon tumour and Meth A sarcoma. In this research, we identified PM-17 as an antitumour compound effective against human pancreatic and gastric cancer cell lines, which induces apoptosis in parallel with autophagy in tumour cells. In addition, PM-17 has more potent antitumour activity than PM-8 *in vivo* (data not shown). It is thought that PM-17 is an active state of PM-8 at inside of the body or cells. PM-8 is reduced by electron transfer system in mitochondria, and becomes PM-17, which is a more cytotoxic agent (Yamase, 2005). Also we think that some of the antitumour activity of PM-8 is derived from reduced species such as PM-17.

From recent reports, the role of autophagy in the maintenance of cellular homoeostasis is becoming clearer. In normal cells, autophagy plays a role in the exclusion of excessive or abnormal proteins and organelles and in the recycling of cellular constituents (Boya *et al*, 2005). In addition, autophagic cell death, through the accumulation of autophagic vacuoles, affects degenerating neurones in some pathological states (Fletcher *et al*, 2000; Larsen *et al*, 2002), participates in retinal degeneration (Guimaraes *et al*, 2003), seals the fate of *Salmonella*-infected macrophages (Hernandez *et al*, 2003), and mediates chemotherapy-induced tumour cell death (Kanzawa *et al*, 2003, 2004; Opipari *et al*, 2004). However, there are few examples of the role of autophagy in physiological and pathological cell death because it is studied mainly based on morphological grounds (Tsujimoto and Shimizu, 2005).

Historically, three types of cell death have been distinguished in mammalian cells on morphological grounds; type 1 cell death better known as apoptosis, type 2 is autophagic cell death, and type 3 is also better known as necrosis. The molecular signalling systems of cell death have been elucidated progressively. In parallel, research has been focused on the connection of signalling between the different models of cell death. For example, in the model of immortalised baby-mouse kidney epithelial cells, which undergo cell death in response to hypoxia by knockdown of the pro-apoptotic proteins (Bax and Bak), however, the overexpression of the antiapoptotic protein Bcl-2 causes a shift from apoptosis to autophagic cell death (Golstein and Kroemer, 2007). In addition, in another model, human breast cancer MCF-7 cells exposed to camptothecin show rapidly induced apoptosis and much slower autophagy. Bid, BH3-only pro-death Bcl-2 family protein, knockout in MCF-7 cells results in suppression of camptothecin-induced apoptosis and a shift of cell death towards autophagy (Lamparska-Przybysz *et al*, 2005). These cases imply that there is a cross network pathway of apoptosis and autophagy in antitumoral cell death.

The results of our study show that PM-17 induces both apoptosis and autophagy in human pancreatic cancer AsPC-1 cells. We examined apoptosis-inductive signalling in tumour cell death, and neither activation of caspase-9 via the mitochondrial system nor activation of caspase-8 via the cytoplasmic system, known as representative apoptotic signalling systems, were confirmed (data not shown). We also verified the activation of caspase-3 and caspase-12 involved in ER stress signalling in preliminary experiments. On the other hand, there was no increase in PI3K(III) or Beclin-1, which were identified as regulatory factors of autophagy. In addition, in our study, AsPC-1 cells were pretreated with 3-MA (inhibitor of PI3K(III) in autophagy signalling) at increasing concentrations from 0 to 1 mM for 30 min before treatment with IC_50_ of PM-17 for 1–24 h, and estimated cell viability using the MTT assay. In this model, the inhibition of autophagy signalling by 3-MA had little effect on cell death induced by PM-17. It is possible that PM-17 activates autophagy through a different unknown route.

As another speculation, autophagy induced by PM-17 treatment does not function as the confrontive death system, but could be a reaction of the clearance system to the accumulation of dysfunctional organelles such as mitochondria or proteins denatured by chemotherapy. This mechanism could also be a rational way to reutilise the components of dying cells as nutrients for remaining cells. At present, we are proceeding to analyse the determination factor of the magnitude of apoptosis and autophagy.

Furthermore, we carried out a toxicity test for PM-17 in mice by haematological examination and serum chemistry (Table 1). In this investigation, blood sera were assessed for BUN, creatinine, total bilirubin, total protein, AST, ALT, and ALP. As a result, there was no significant difference between the control and PM-17 groups. The results showed that there was no impaired renal or hepatic function, nor haematocytes in our administration model of PM-17.

In conclusion, PM-17 may be useful as a novel effective reagent in treating pancreatic cancers, which generally have poor outcomes. We plan to investigate the induction mechanism of tumour cell death in detail, especially how PM-17 turns the cell death programme on, and continue research for *in vivo* models of antitumour therapy and the development of novel drug-delivery systems using PM-17 (Yanagie *et al*, 1997, 2004, 2006).

## Figures and Tables

**Table 1:** Haematological examination and blood chemistry

**Group**	**WBC ( × 10^3^ mm^−3^)**	**RBC ( × 10^5^ mm^−3^)**	**HGB (g dl^−1^)**	**HCT (%)**	**MCV (*μ*m^3^)**	**MCH (pg)**	**MCHC (%)**	**PLT ( × 10^3^ mm^−3^)**
Control	2.53±1.42	10.06±0.42	17.1±0.8	48.5±2.3	48.2±0.5	17.0±0.2	35.2±0.3	924±69
PM-17 500 *μ*g	4.08±1.23	10.18±0.32	17.1±0.7	48.9±1.8	48.0±0.5	16.8±0.2	35.0±0.3	750±148
PM-17 1000 *μ*g	4.51±2.54	9.76±0.16	16.7±0.3	46.4±0.7	47.6±0.7	17.1±0.2	36.0±0.7	1075±337
Negative control	3.54±0.76	10.08±0.25	17.2±0.3	49.6±1.1	49.2±0.2	17.1±0.1	34.7±0.1	805±333

	**Differential leukocyte counts (%)**							
**Group**	**NEUT**	**LYMPH**	**MONO**	**EOSN**	**BASO**	**LUC**	**Reticulocyte (%)**	
Control	38.5±10.2	46.3±7.7	1.9±0.5	12.8±5.9	0.2±0.1	0.3±0.1	3.7±0.3	
PM-17 500 *μ*g	26.3±8.4	60.7±11.6	2.1±0.5	10.2±5.8	0.2±0.1	0.5±0.3	3.5±0.4	
PM-17 1000 *μ*g	30.5±6.0	56.3±17.3	2.0±0.4	10.6±12.0	0.1±0.1	0.4±0.2	3.5±0.6	
Negative control	20.6±1.8	68.2±7.8	1.6±0.1	9.2±6.4	0.3±0.1	0.4±0.2	4.0±0.5	

								
**Group**	**Total protein (g dl^−1^)**	**AST (U l^−1^)**	**ALT (U l^−1^)**	**ALP (U l^−1^)**	**Total bilirubin (mg dl^−1^)**	**BUN (mg dl^−1^)**	**Creatinine (mg dl^−1^)**	
Control	5.33±0.68	653±627	144±133	288±28	0.02±0.01	23.0±3.6	0.12±0.02	
PM-17 500 *μ*g	4.94±0.21	236±162	117±117	263±21	0.02±0.02	19.4±2.6	0.10±0.02	
PM-17 1000 *μ*g	4.97±0.23	301±93	96±66	250±33	0.01±0.02	23.9±1.0	0.13±0.02	
Negative control	4.76±0.08	337±114	271±156	357±40	0.00±0.00	29.6±0.4	0.13±0.02	

Data indicate mean±s.d. per group. There is no significant difference between PM-17-treated groups and control.
